# AI-Driven Motion Capture Data Recovery: A Comprehensive Review and Future Outlook

**DOI:** 10.3390/s25247525

**Published:** 2025-12-11

**Authors:** Ahood Almaleh, Gary Ushaw, Rich Davison

**Affiliations:** School of Computing, Newcastle University, Newcastle upon Tyne NE1 7RU, UKrichard-gordon.davison@newcastle.ac.uk (R.D.)

**Keywords:** 3D human, motion capture data, motion recovery, gap reconstruction, survey, review, deep learning, neural networks, GANs, GNNs, biomechanics, human movement analysis, transformers

## Abstract

This paper presents a comprehensive review of motion capture (MoCap) data recovery techniques, with a particular focus on the suitability of artificial intelligence (AI) for addressing missing or corrupted motion data. Existing approaches are classified into three categories: non-data-driven, data-driven (AI-based), and hybrid methods. Within the AI domain, frameworks such as generative adversarial networks (GANs), transformers, and graph neural networks (GNNs) demonstrate strong capabilities in modeling complex spatial–temporal dependencies and achieving accurate motion reconstruction. Compared with traditional methods, AI techniques offer greater adaptability and precision, though they remain limited by high computational costs and dependence on large, high-quality datasets. Hybrid approaches that combine AI models with physics-based or statistical algorithms provide a balance between efficiency, interpretability, and robustness. The review also examines benchmark datasets, including CMU MoCap and Human3.6M, while highlighting the growing role of synthetic and augmented data in improving AI model generalization. Despite notable progress, the absence of standardized evaluation protocols and diverse real-world datasets continues to hinder generalization. Emerging trends point toward real-time AI-driven recovery, multimodal data fusion, and unified performance benchmarks. By integrating traditional, AI-based, and hybrid approaches into a coherent taxonomy, this review provides a unique contribution to the literature. Unlike prior surveys focused on prediction, denoising, pose estimation, or generative modeling, it treats MoCap recovery as a standalone problem. It further synthesizes comparative insights across datasets, evaluation metrics, movement representations, and common failure cases, offering a comprehensive foundation for advancing MoCap recovery research.

## 1. Introduction

Motion capture (MoCap) data, a digital representation of human motion recorded by professional systems, has become integral to various fields such as 3D animation, film production, sports analysis, medical diagnostics, biomechanics, human-computer interaction, virtual reality, and robotics [1]. Its ability to capture and replicate complex movements makes it invaluable for research and practical applications [2]. Despite their utility, MoCap data often suffers from gaps and noise due to occlusions, malfunctions, and difficulties in capturing complex or rapid movements. Missing or corrupted data can lead to unnatural animations and unreliable analyses, underscoring the importance of robust recovery techniques [3]. Accurate reconstruction of this data is essential to ensure smooth and realistic motion representation in applications such as animation, healthcare, and biomechanical studies. Research in MoCap data analysis has grown significantly, focusing on various aspects, including motion prediction, completion filters, multimodal data, and style transfer. For instance, human motion prediction aims to generate future frames to create new motion sequences but does not directly address gaps in existing data [4]. Similarly, studies on denoising techniques and generative models emphasize noise reduction and multisensory data handling [2,5], while research on style transfer focuses on enhancing visual consistency [6]. However, these studies fall short of comprehensively reviewing methods designed to recover incomplete or corrupted motion capture data, leaving a significant gap in the literature [2,4,5,7,8,9,10,11]. While prior reviews have focused on related topics such as motion prediction, denoising, or style transfer, none have comprehensively addressed the specific challenge of recovering missing or corrupted MoCap data. This paper addresses this gap by providing the first survey of methods for MoCap data recovery. Systematically examines traditional, deep learning, physics-based, and filter-based approaches, organizing them into a structured taxonomy that reflects the evolution of recovery techniques. By conducting a comparative analysis, this review evaluates the strengths and limitations of existing methods and identifies opportunities for future research to improve their reliability and adaptability. Our contribution is distinct in its focus on MoCap recovery, offering researchers a consolidated reference that complements, rather than overlaps, existing surveys and provides valuable guidance for advancing the field. It is structured as follows: Section 2 introduces the basic concepts of motion capture technologies, data types, and challenges, providing a foundational understanding of the field. Section 3 describes the survey methodology, including the literature search process, inclusion and exclusion criteria, and data extraction and analysis procedures. Section 4 presents the taxonomy of motion capture data recovery techniques, classifying approaches into data-driven, non-data-driven, and hybrid methods. Section 5 provides a comprehensive and critical analysis of motion capture data recovery techniques. This section is particularly valuable, as it synthesizes all the information from the identified studies collected for this survey. Examine their purposes, methodologies, datasets, movement representation types, evaluation metrics, and the most effective techniques in the field. In addition, it reviews citation patterns and study designs, identifies trends, and discusses the limitations of current approaches while offering directions for future research. Finally, Section 6 concludes the paper, summarizing the findings and providing recommendations for MoCap data recovery methods.

## 2. Basic Concepts of Motion Capture Systems

This section explores the fundamental aspects of MoCap systems, including the primary technologies, the data types they generate, and the key challenges in ensuring data accuracy and completeness. This provides the basis for understanding the MoCap recovery techniques discussed in later sections. MoCap systems are broadly categorized into marker-based and markerless systems, each with distinct advantages and challenges depending on the technology and data they produce [12,13,14]. Marker-based systems rely on physical markers attached to key body points, such as joints or segments of interest. These markers are tracked by an array of cameras, often using infrared technology, to capture precise three-dimensional (3D) positions as the subject moves. A common marker-based approach is optical motion capture, which uses reflective markers illuminated by infrared cameras. This method is widely used in biomechanics, sports science, and animation for film and gaming due to its high accuracy. However, marker-based systems are not without limitations. They require complex setup and calibration in controlled environments, which can be time-consuming and resource-intensive. Furthermore, occlusions, when a marker is obscured from the camera’s view due to body positioning, can lead to data gaps or inaccuracies, especially in scenarios involving dynamic movements or multiple subjects [15]. In contrast, markerless systems capture motion data without physical markers, relying instead on visual and depth-based technologies. These include RGB-D cameras, which capture colour and depth information, and depth sensors using structured light or time-of-flight technology to measure distance. Markerless systems have gained popularity due to advances in computer vision, enabling greater flexibility and easier setup. They are particularly useful in naturalistic or outdoor environments where markers are impractical. Participants also benefit from the reduced burden of wearing markers. However, markerless systems face challenges in accuracy due to their greater vulnerability to occlusions and noise. For example, complex or rapid movements may obscure parts of the body from sensors, resulting in incomplete data [16]. These limitations often require advanced processing techniques to fill in missing data and correct inaccuracies caused by noise [14]. Marker-based and markerless systems produce several types of data, each with implications for motion recovery techniques. Common outputs include 3D joint coordinates, central to joint-based motion analysis, and marker trajectories, which capture the detailed paths of markers over time and are widely used in marker-based systems for high-resolution motion data [11,13,17]. Markerless systems often generate point clouds, offering real-time 3D representations of the subject’s movement, and depth maps, which provide pixel-level depth information valuable for reconstructing skeletal movements [18]. Skeletal representations, which infer the subject’s skeletal structure from depth or point-cloud data, are commonly used to model body posture and movement. Each data type poses unique challenges; for instance, point clouds and depth maps may require substantial cleaning and interpolation, whereas skeletal models are prone to inaccuracies in joint-angle calculations. To develop effective motion recovery methods, it is essential to understand the unique properties and complexities of various motion data sources. Another crucial aspect is the array of challenges that MoCap systems face, including noise, occlusions, marker misplacement, and data corruption [19,20,21]. In marker-based systems, common problems include occlusion, where markers are occluded from the camera view, and misplacement or swapping of markers, both of which can result in errors in motion data. Additionally, false signals, known as ghost markers, and calibration errors can introduce noise and lead to jittery or inaccurate tracking, particularly in dynamic scenarios such as sports or dance. While eliminating the need for markers, markerless systems lack absolute positional accuracy and are prone to visual interference from shadows, reflections, and changes in lighting. They also reduce accuracy in high-speed movements. Missing markers, incomplete trajectories, and noisy data create gaps that affect analysis. Methods such as frame reconstruction, noise reduction, and data recovery are essential for improving MoCap accuracy and reliability in dynamic applications.

## 3. Survey Methodology

This review systematically analyzes motion capture data recovery techniques, focusing on their classification, evaluation, and comparative analysis. The methodology is based on a structured literature review, drawing on insights from a detailed matrix comprising 40 studies published between 2016 and 2024.

### 3.1. Literature Search Process

**Figure 1:**
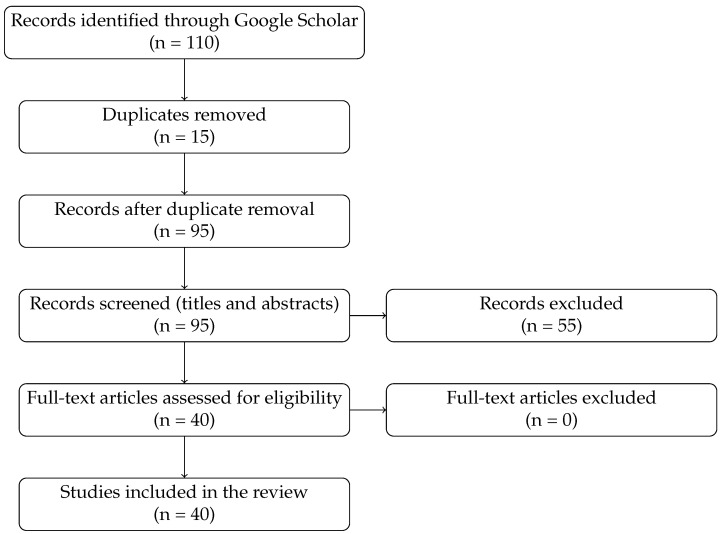
PRISMA flow diagram for study selection.

A comprehensive literature search was conducted using Google Scholar, employing combinations of keywords such as “motion capture recovery,” “data reconstruction,” and “MoCap gap-filling,” as well as terms such as “recovery,” “completion,” and “deep learning.” This search initially found 110 studies. After removing duplicates and screening for relevance, 40 studies met the inclusion criteria and were analyzed in detail. These studies are the basis for the comparative matrix in Section 3.3. To enhance transparency, a PRISMA-style flow diagram (Figure 1) has been added to illustrate how the initial set of 110 records was progressively refined to the final 40 studies included in this review.

### 3.2. Inclusion and Exclusion Criteria

To ensure relevance and focus, studies were included based on the following criteria:

- Inclusion Criteria: Studies published between 2016 and 2024 in peer-reviewed journals or conference proceedings were considered. They needed to address motion capture data recovery, reconstruction, or gap-filling, emphasising deep learning methods or traditional computational techniques.
- Exclusion Criteria: Studies that focus exclusively on synthetic data without practical application, research centred on motion capture data generation from other modalities (e.g., text or images), and techniques aimed solely at style transfer or human activity recognition were excluded. These topics were considered outside the scope of MoCap data reconstruction.

### 3.3. Data Extraction and Analysis

A detailed matrix was developed to systematically document and analyze key attributes of 40 studies, enabling a comprehensive review of MoCap recovery and reconstruction techniques. This structured approach facilitated comparative analysis and the identification of trends across methodologies. The matrix included several critical attributes to ensure a thorough evaluation. First, it documented the number of citations for each study to gauge their impact within the field. Second, it categorized the study designs to understand the methodologies employed in the research. Third, it analyzed the objectives of the studies, focusing on the specific aspects of MoCap reconstruction addressed, such as gap-filling, noise reduction, or cross-domain recovery. Additionally, the matrix recorded the methodologies utilized for MoCap recovery and the benchmark datasets employed, which served as a basis for evaluating performance. Evaluation metrics, including accuracy, computational efficiency, and robustness, were used to assess the effectiveness of these techniques. Findings and limitations were also reported, highlighting the observed outcomes and challenges, including difficulties in handling complex motion patterns. Furthermore, the type of motion data analyzed, whether joint-based, marker-based, or 3D skeletal representations, was documented to understand the adaptability of the techniques to various forms of MoCap data. By organizing these attributes into a comprehensive matrix, the framework provided a detailed breakdown of the methodologies and their performance, facilitating a deeper understanding of the trends, strengths, and limitations of existing MoCap recovery techniques.

## 4. Taxonomy of Motion Data Recovery Techniques

**Figure 2:**
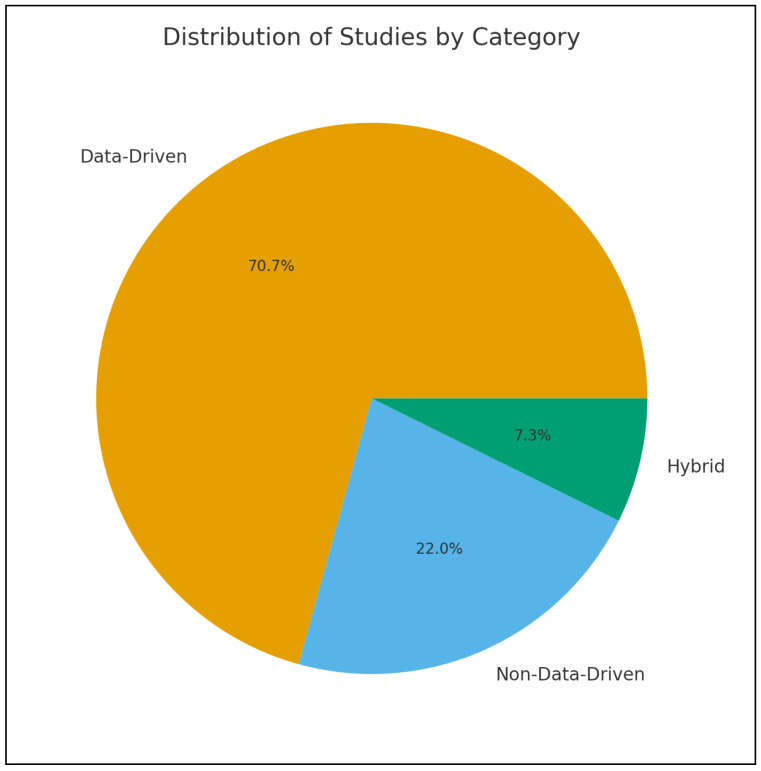
Proportional distribution of the reviewed studies by methodological category, showing the dominance of data-driven approaches over non-data-driven and hybrid methods.

The recovery of motion capture data is critical to improving the accuracy and applicability of MoCap systems. Missing or corrupt data can significantly undermine their utility, making effective reconstruction techniques essential. Researchers have used various classification criteria to categorize methodologies. A widely recognized classification distinguishes between deep learning and traditional methods, reflecting the evolution from rule-based algorithms to flexible, data-driven models capable of capturing complex, non-linear patterns. For example, Holden et al. classify methods based on their underlying methodologies, transitioning from rule-based interpolation models to adaptive machine-learning approaches [22]. Lyu et al. classified the methods for Human Motion prediction into Human Pose Representations, Network Structure Design, and Prediction Targets [4]. Pose representation encompasses physical models based on skeletal data and kinematics, as well as mathematical models based on graphs and trajectories. Network structures involve RNNs, CNs, and GANs to handle temporal, spatial, and probabilistic dependencies. Prediction targets include deterministic models for single outcomes and probabilistic models for multiple plausible sequences. Domain-specific applications represent another common classification. In rehabilitation, computational methods are grouped into discrete movement scoring, rule-based, and template-based categories [23]. Camargo et al. explored biomechanics, comparing their physics-based inverse kinematics approach to other techniques for gait analysis [24]. Emerging classifications highlight hybrid techniques, combining statistical or physics-based methods with deep-learning models. For instance, Martini et al. proposed categories such as General Purpose Filters for data smoothing, State Observers (e.g., Kalman Filters) for motion estimation, Dimensionality Reduction methods (e.g., PCA), Deep Neural Networks (e.g., Denoising Autoencoders) and Hybrid Approaches such as graph Neural Networks combined with Autoencoders [5]. To reflect the progression of methodologies and trends in the field, this review proposes a taxonomy that classifies human motion recovery methods into three approaches: non-data-driven, data-driven, and hybrid, based on the underlying architecture. Figure 2 illustrates the proportional distribution of the reviewed studies across the three methodological categories: non-data-driven, data-driven, and hybrid approaches. The figure shows that data-driven methods dominate the field, whereas non-data-driven and hybrid techniques constitute much smaller proportions. This distribution highlights the strong shift toward AI-based solutions in MoCap recovery research.Each category includes specific methodologies tailored to address various recovery challenges, balancing computational complexity, accuracy, and robustness in different ways. In the following section, a comparative analysis of these techniques will be performed to evaluate their effectiveness and applicability. Table 1 provides a comparison between the different categories. This taxonomy is well suited for MoCap recovery because it reflects the computational strategies used to reconstruct missing or corrupted spatio-temporal data. Unlike prior classifications based on tasks or modalities, recovery methods naturally group into three methodological categories: non-data-driven approaches built on deterministic mathematical or physical rules; data-driven approaches that learn nonlinear motion patterns from data; and hybrid approaches that combine model-based constraints with learning-based flexibility. Organizing the literature around these paradigms resolves ambiguities in earlier schemes and provides a coherent recovery-focused framework that offers a comprehensive view of MoCap reconstruction techniques.

### 4.1. Non-Data-Driven Methods

These foundational techniques rely on predefined mathematical, physical, or optimization based rules to reconstruct missing or corrupted motion capture (MoCap) data. They are computationally efficient and particularly effective in scenarios involving straightforward or predictable motion patterns. Traditional approaches like cubic or linear interpolation are simple yet heavily reliant on manual user input and assumptions about the surrounding data’s accuracy. While these methods are time-intensive and lack robust data-driven foundations, they provide interpretable results and suit scenarios, prioritizing reliability and simplicity [23,24]. Advancements in matrix completion methods have brought significant improvements. For example, Wang et al. (2016) proposed a low-rank matrix recovery approach that represents motion sequences as corrupted matrices, recovering clean data by minimizing the nuclear norm and normalizing the error matrix noise [25]. This technique effectively corrects complex skeleton data without additional training, making it particularly useful for occlusion and noise recovery. A fully automatic method for filling gaps in motion capture trajectories employs least-squares optimization and Kalman Filter to reconstruct missing marker positions without requiring manual intervention or training data [26]. Kalman filters, recursively estimate unknown variables, as demonstrated by Gomes et al. (2021), who integrated these filters with optimization strategies to efficiently handle noisy and incomplete datasets [26]. Non-convex optimization methods, which tackle problems with multiple local optima, outperform traditional methods in recovering highly corrupted datasets. Low-rank and sparse optimization methods are critical for decomposing motion capture data into structured motion patterns and noise. Kamali et al. (2020) employed this approach for gait analysis, showcasing its robustness for large and noisy datasets [27]. Dimensionality reduction models, like Principal Component Analysis (PCA), transform high-dimensional data into a lower-dimensional space, maintaining essential motion characteristics. Li et al. (2020) combined PCA with interpolation to reconstruct missing motion data, ensuring smooth transitions and structural integrity [28]. Robust PCA (RPCA), which separates low-rank and sparse components, offers resilience to outliers. Raj and George (2023) used RPCA with pairwise hierarchical and trajectory constraints to achieve smooth and spatially consistent skeletal movements [45]. Inverse kinematics-based methods have also proven highly effective. Camargo et al. (2020) developed an automated gap-filling method that iteratively minimizes kinematic errors, significantly reducing manual intervention while enhancing trajectory accuracy [24]. Statistical and kinematic-based approaches also leverage probabilistic principles and physical constraints to ensure realistic motion outputs. Probabilistic Model Averaging (PMA), as employed by Tits et al. (2018), combines predictions from multiple models and integrates skeleton constraints to refine motion trajectories, providing robust solutions for marker reconstruction and smoothing [29]. Non-data-driven models offer robust, interpretable, and computationally efficient motion capture (MoCap) data processing solutions. Optimization-based techniques, such as Kalman filters and low-rank matrix recovery, effectively address missing data and noise in motion sequences by solving well-defined mathematical problems under constraints. Dimensionality reduction methods, including Principal Component Analysis (PCA) and Robust PCA (RPCA), simplify complex datasets while preserving essential motion features, enabling efficient recovery and reconstruction of motion patterns. Statistical and kinematic approaches, such as Probabilistic Model Averaging (PMA) and inverse kinematics, ensure anatomically plausible motion recovery by incorporating domain-specific constraints like joint limits and physical plausibility. Collectively, these methods form a comprehensive toolkit for enhancing the accuracy and reliability of MoCap systems in various applications. These approaches are widely utilized in domains where deterministic models and computational efficiency are paramount. In biomechanics, these methods enable precise motion smoothing, noise removal, and accurate gait analysis [24]. In robotics, they ensure reliable trajectory predictions for structured environments, enabling precise and repeatable control [15]. Despite their strengths in handling smaller datasets and simpler motion patterns, these methods face significant challenges with large-scale datasets, complex non-linear motion, and extensive occlusions [46]. Moreover, their inability to integrate real-world biomechanical constraints, such as consistent bone lengths or rigid-body dynamics, limits their accuracy and applicability in scenarios requiring high fidelity and biomechanical realism. These limitations underscore the need for hybrid or data-driven augmentations to overcome the constraints inherent to non-data-driven approaches. By integrating these methods with advanced computational techniques, such as machine learning and multimodal sensor data, researchers can enhance their robustness and adaptability to tackle more complex and dynamic motion recovery challenges. Such hybrid models hold the potential to combine the interpretability of non-data-driven methods with the flexibility and precision of data-driven innovations, addressing the evolving demands of modern motion capture systems.

### 4.2. Data-Driven Methods

Data-driven approaches rely on patterns and relationships learned directly from data, often through machine learning or deep learning, without requiring handcrafted rules, explicit mathematical models, or additional algorithmic enhancements [10,31,34,35]. These methods represent the forefront of innovation in motion capture recovery, leveraging advanced computational techniques to extract meaningful insights from complex datasets. Offering adaptive and scalable solutions, they effectively reconstruct intricate, non-linear motion patterns often challenging for traditional approaches [47,48,49]. Recent advances have introduced diverse methodologies, including neural networks, attention-based models, autoencoders, graph neural networks (GNNs), generative models, and traditional machine-learning techniques. Each method offers unique capabilities: neural networks capture intricate data relationships, attention-based models dynamically prioritize critical information, autoencoders learn compact data representations, GNNs excel with graph-like structures, and generative models create realistic motion sequences. This section reviews these techniques, emphasizing their contributions and advancements, as identified in the collected studies.

#### 4.2.1. Neural Network-Based Models

Neural network-based models are fundamental for processing motion capture data because they can model complex nonlinear relationships and capture temporal dependencies. These capabilities make them particularly effective for tasks such as recovering missing data. Leveraging deep learning architectures like recurrent neural networks (RNNs), convolutional neural networks (CNNs), and transformers, these models handle sequential and spatial data with high precision and efficiency. For instance, Feedforward neural networks (FFNNs) process data in a unidirectional flow, making them well-suited for static feature learning. Holden (2018) demonstrated that FFNNs effectively remove noise from motion data while preserving its integrity, ensuring minimal distortion [22]. Similarly, RNN variants, particularly Long Short-Term Memory Networks (LSTMs), are widely employed for handling sequential data. These models excel at capturing long-term dependencies in motion data. Studies by Kucherenko et al. (2018) and Zhu (2020) highlight LSTMs’ effectiveness in reconstructing missing markers and learning spatial-temporal relationships and show how LSTMs outperform interpolation-based methods [47,50]. Enhanced models, such as Zhu and Cai’s LSTNet, integrate temporal and spatial modelling, demonstrating robust performance and low reconstruction error, especially for periodic motion sequences [51].

#### 4.2.2. Traditional Machine Learning Approaches

Machine learning (ML) techniques are widely used for analyzing and processing motion capture data, owing to their ability to learn patterns from data and make predictions or classifications [13]. ML models, such as regression, decision trees, and clustering, provide interpretable and computationally efficient solutions for straightforward tasks, complementing more complex neural network-based approaches. For instance, Skurowski and Pawlyta (2024) evaluated tree-based regression methods, including M5P and ensemble models, highlighting their effectiveness in reconstructing long gaps in motion capture data without requiring extensive training datasets. Such methods balance accuracy and efficiency for specific data recovery tasks [52]. Alemi et al. (2019) [49] reviewed data-driven techniques for human motion animation, emphasizing the role of machine learning in MoCap data preprocessing, recovery, and evaluation. Their review discussed the integration of ML models in the animation pipeline, highlighting their capability to address challenges like missing markers, motion noise, and efficient data analysis. Data-driven approaches offer exceptional performance and scalability in handling complex motion patterns, making them indispensable for modern applications. By leveraging large datasets, these methods eliminate the need for handcrafted features and can adapt to diverse scenarios. They are widely applied across animation, gaming, sports, healthcare, robotics, and VR/AR domains. Techniques like Generative Adversarial Networks (GANs) and transformers enable realistic motion reconstruction, while Graph Neural Networks (GNNs) refine athlete movements and enhance human-robot interactions. In healthcare, models like UBiLSTM contribute significantly to rehabilitation efforts, while attention-based transformers create lifelike VR experiences. Furthermore, accurate motion recovery has biomechanics, film production, surveillance, and education applications. Despite their advantages, data-driven approaches face notable challenges. Their reliance on high-quality datasets can limit generalization to unseen motion patterns, and computational inefficiencies make real-time applications difficult. Overfitting is another concern, as models trained on specific datasets may struggle to handle real-world variability. However, these challenges have not overshadowed the transformative impact of data-driven techniques, which excel at learning intricate spatial-temporal relationships in motion data. By overcoming dataset diversity, efficiency, and generalization challenges, data-driven approaches are poised to revolutionize motion capture recovery and expand into autonomous systems and immersive live applications.

### 4.3. Hybrid Approaches

Hybrid approaches combine advanced computational techniques to address the challenge of recovering missing or corrupted markers in human motion capture data. They leverage machine learning models and traditional algorithmic frameworks for enhanced accuracy and efficiency. One notable method is the locally weighted PCA regression with sparsity constraints. This approach efficiently estimates missing data by weighting local neighbourhoods of the dataset and applying principal component analysis (PCA) to preserve underlying motion structures [42]. The method excels in high estimation accuracy, numerical stability, and real-time applicability, making it a reliable option for motion data recovery. Another innovative technique employs an attention-based Long Short-Term Memory (A-LSTM) network with a least-squares (LS) constraint. The model effectively captures temporal dependencies in motion sequences by integrating attention mechanisms, while the LS constraint ensures mathematical rigour and stability. This dual framework effectively combines deep learning with optimization techniques to improve the robustness and accuracy of motion recovery compared to existing methods, offering significant advancements in handling diverse motion capture scenarios [43]. Finally, an efficient and scalable framework introduces a GPU-based parallel kd-tree for nearest-neighbour retrieval that integrates attention mechanisms with least squares. This data-driven method processes missing data by dynamically searching for the most relevant motion patterns in high-dimensional spaces. The framework demonstrates superior performance over state-of-the-art techniques, particularly on benchmark datasets, due to its ability to handle large-scale computations rapidly and effectively [44]. Hybrid approaches for motion data recovery combine machine learning and traditional algorithms to balance accuracy, efficiency, and adaptability. They leverage advanced techniques like attention mechanisms, graph-based models, and generative frameworks to reconstruct complex motion patterns, supporting diverse biomechanics, gaming, robotics, and VR/AR applications. These methods are effective for patient monitoring, performance analysis, and real-time human-robot interactions. However, challenges such as computational overhead, reliance on labelled data, and limited generalizability remain. Addressing these requires architecture, optimization, and dataset diversity innovations to enhance scalability and robustness. This taxonomy reflects current trends in the literature, providing a structured framework to evaluate and compare MoCap recovery techniques. By examining methodologies, emerging trends, application domains, and the strengths and limitations of each category, researchers can gain a comprehensive understanding of MoCap data recovery and its future potential. These categories highlight the breadth of innovation in MoCap recovery, as demonstrated by the analysis of collected studies and their contributions to the field. Each category represents a significant step forward in addressing the challenges of reconstructing and refining human motion data, paving the way for continued advancements.

## 5. Comprehensive Analysis of Motion Recovery Techniques: Trends and Future Directions

This section analyses the collected studies to identify emerging trends, evaluate employed techniques, and determine the most utilized methods. It examines the strengths and limitations of each approach, explores evolving methodologies, and discusses their implications for the domain while uncovering research opportunities to advance motion capture recovery techniques. Furthermore, the analysis delves into the most frequently used datasets, evaluation methods, and metrics, offering a holistic understanding of the standards and benchmarks shaping the field.

### 5.1. Techniques Among Purposes

An analysis of human motion recovery studies reveals three primary objectives: noise removal, gap filling, and quality enhancement. These objectives address key challenges in motion capture data recovery, forming interconnected research areas that contribute to advancements in MoCap technologies. Examining the techniques used in these studies makes it clear that different methodologies are applied depending on the specific purpose, with some approaches combining multiple objectives for comprehensive solutions (see Table 2). Gap filling focuses on reconstructing missing markers caused by occlusion or corruption. Neural networks like LSTM, GRU, and BiLSTM dominate due to their ability to model temporal and spatial dependencies. At the same time, classical methods such as Locally Weighted PCA Regression and Regression Trees provide efficient interpolation. Optimization techniques, including Low-Rank Matrix Recovery and kd-tree structures, further support structured and scalable recovery. While Noise removal techniques refine motion trajectories affected by disturbances. Recurrent Neural Networks (RNNs) and attention mechanisms, such as Bidirectional RNN with Attention, prioritize important features for denoising. Graph Neural Networks (GNNs) are also widely used for spatial-temporal refinement, reflecting the trend toward adaptive and precise noise handling. Combined noise removal and gap filling approaches use advanced models like UBiLSTM Autoencoders and Graph Normalizing Flow. These methods integrate spatial-temporal reasoning to handle complex recovery tasks. Similarly, quality enhancement studies focus on generative models like GANs to augment data, alongside DenseNet and SVM, to improve motion data quality. Some studies address all three objectives: noise removal, gap filling, and quality enhancement, integrating deep learning and modular approaches like Divide-and-Conquer Strategies to achieve holistic solutions. Techniques like Spatial-Temporal Graph Motion Glow exemplify simultaneous noise reduction, gap filling, and quality enhancement advancements. These methods underscore the growing reliance on hybrid, generative, and adaptive models to meet the diverse needs of MoCap data recovery, pushing the boundaries of technology in this field. Other studies focus on specific combinations of objectives, such as noise removal and quality enhancement or quality enhancement and gap filling. For noise removal and quality, attention-based neural networks and Bidirectional RNNs with Attention effectively refine noisy data while improving its overall quality. Techniques like GANs and DenseNet are also used to generate high-quality augmented datasets. For quality enhancement and gap filling, transformers, such as Attention-Based Transformers, and generative models like Temporal Convolutional GANs (TCGAN), play a crucial role in seamless reconstruction and improvement. Optimization methods, including Low-Rank Matrix Restoration with Sparse Priors and Kalman Filters, ensure robustness and accuracy in these tasks. The analysis reveals that neural networks, attention mechanisms, and graph-based models are the dominant techniques in multiple objectives. Hybrid approaches and generative models are increasingly shaping the field, offering robust solutions to address challenges in MoCap data recovery. Across these categories, several important limitations and failure cases arise that shape the practical performance of MoCap recovery techniques. Non-data-driven methods frequently fail when motion complexity or occlusion increases; interpolation-based techniques oversmooth trajectories, low-rank models struggle under non-linear motions, and inverse kinematics often diverge when markers are heavily occluded or incorrectly labeled. Data-driven approaches introduce different constraints: deep networks tend to overfit benchmark datasets such as CMU and HDM05, leading to poor generalization to new subjects, clothing, marker configurations, or multi-person scenes. Failure cases commonly include transformer degradation when recovering long gaps, LSTM saturation with irregular or sparse data, and GNN breakdowns when joint connectivity assumptions are violated. GAN-based models may suffer from mode collapse, producing plausible but biomechanically inconsistent motion segments. Hybrid models reduce some of these issues but still face instability when balancing learned patterns with kinematic or physics-based constraints; for example, weak biomechanical priors can result in bone-length drift, joint-angle discontinuities, or physically implausible trajectories. These limitations underscore the need for future MoCap recovery frameworks to incorporate stronger biomechanical reasoning, more diverse datasets, and robustness to real-world noise and occlusion conditions. When comparing model families, recurrent networks such as LSTMs excel at modelling smooth temporal dependencies in short to medium-length sequences but degrade when gaps become long or when the motion contains abrupt transitions. Transformers, by contrast, can capture global temporal relationships more effectively due to their attention mechanism, but they require substantially larger datasets and are computationally more expensive; they also exhibit instability when recovering extended missing intervals or when training data lack diversity. Graph-based networks (GNNs) leverage skeletal topology to enforce relational structure, making them highly effective for preserving joint connectivity, yet they are sensitive to errors in skeleton definitions and fail when joint adjacency assumptions are violated. GAN-based architectures generate realistic trajectories but suffer from mode collapse and provide limited biomechanical control, making them unreliable for recovery tasks. Traditional PCA or low-rank methods perform well for structured or low-complexity motions but break down under nonlinear dynamics, significant noise, or high occlusion. Hybrid approaches mitigate some of these weaknesses by combining learned spatial–temporal priors with physical or kinematic constraints, although they may become unstable when the constraints contradict the learned predictions.

### 5.2. Datasets

The selection of datasets is a critical factor influencing the design, evaluation, and generalizability of methodologies in human motion capture (MoCap) research. A systematic review of dataset usage reveals a predominant reliance on benchmark repositories, synthetic datasets, and domain-specific real-world datasets, each facilitating distinct research objectives and experimental configurations. Among these, the Carnegie Mellon University Motion Capture Database (CMU) [65] emerges as the most widely adopted resource, cited in 23 studies, accounting for approximately 57% of dataset references. Its comprehensive repository encompasses a broad spectrum of motion sequences, rendering it suitable for diverse recovery and reconstruction tasks. The HDM05 Motion Database [66] is another popular choice, offering high-quality motion data specifically curated for human movement analysis. Likewise, the Human3.6M Dataset [67], which includes complex motions and detailed 3D skeleton annotations derived from marker-based capture, is frequently used for algorithm benchmarking and fine-grained analysis. Synthetic datasets provide controlled environments ideal for testing algorithms under varying conditions. For instance, the SMPL + H synthetic dataset focuses on the synthesis of body and hand movements [37,39]. Other synthetic datasets, incorporating noise and data augmentation, allow researchers to assess model robustness against different types of data gap. A smaller number of studies use specialized real-world datasets to meet specific research needs. These include proprietary game studio datasets for applications in digital entertainment, labeled marker point cloud datasets for high-precision motion analysis, and culturally specific datasets such as the Southeast Asian Traditional Dance Dataset, which supports niche motion capture recovery applications [28]. Overall, the distribution of dataset usage reflects a reliance on well-established benchmarks like CMU and HDM05 for generalizability and model training, while synthetic and specialized datasets are employed for targeted evaluations, and domain-specific investigations.A critical limitation across current recovery research is the heavy dependence on CMU and HDM05 datasets, which introduces several structural biases. These datasets predominantly feature single-person motions recorded in controlled indoor environments with consistent lighting, minimal occlusion, and constrained clothing styles. As a result, recovery models trained on these datasets do not encounter realistic challenges such as multi-person interactions, self-occlusions, loose or complex clothing, rapid limb-to-limb occlusions, or cluttered environments. Moreover, the standardized marker layouts in CMU and HDM05 mask practical real-world issues such as marker misplacement, marker swapping, and dropout. These limitations restrict the generalizability of recovery techniques and help explain the sharp performance drop observed when applying trained models to sports, clinical, outdoor, or markerless scenarios. Addressing dataset diversity is therefore essential for developing robust, real-world MoCap recovery methods. An important future direction highlighted across the reviewed literature is the integration of explicit biomechanical constraints into recovery models. Current learning-based approaches often produce kinematically valid but biomechanically inconsistent motion, particularly under severe occlusion or long missing intervals. Incorporating joint-angle limits, torque constraints, and bone-length invariance can ensure anatomical plausibility and prevent artifacts such as hyperextension, discontinuous joint trajectories, or bone-length drift. Furthermore, musculoskeletal dynamics—including muscle activation patterns, force generation, and energy constraints—offer a rich source of information that can improve the realism and physical validity of reconstructed motion. Embedding these principles into neural architectures, either through physics-informed loss functions, differentiable biomechanical simulators, or hybrid optimization layers, represents a promising direction for developing recovery methods that are both accurate and physiologically grounded.

### 5.3. Types of Human Movement Representation

The studies reviewed in this research offer critical insights into the diverse data representations used for capturing human movement. These representations are primarily categorized into skeleton data (joint coordinates), marker-based data (3D marker trajectories), and hybrid approaches that integrate both. Each representation presents unique advantages tailored to the demands of specific motion capture tasks. Skeleton data, which captures the 3D positions of human body joints, is a widely used representation due to its compactness and suitability for high-level motion analysis [25,33,52,55]. This data type is increasingly used in applications like pose estimation, gait analysis, and motion prediction, particularly where marker-less systems are employed. Such systems rely on vision-based approaches or AI-driven estimation to reconstruct joint coordinates without requiring physical markers, making them accessible and scalable for real-world scenarios In contrast, some studies used Marker-based data, offering a more granular representation by tracking the trajectories of physical markers attached to the body [3,37,42,43]. This representation is essential in domains requiring high precision, such as biomechanics, animation, and robotics. Marker trajectories capture detailed motion patterns, including subtle nuances, making them invaluable for facial expression analysis or sports biomechanics applications. However, marker-based systems are not without limitations. Marker occlusion, misplacement, and hardware costs often present barriers to widespread use. Studies employing Hybrid approaches combining skeleton data and marker trajectories are increasingly common as they leverage the complementary strengths of both representations. By integrating skeleton data’s efficiency with marker data’s precision, For instance [40,55]. Current trends indicate a shift toward skeleton-based data, driven by advancements in computer vision and machine learning that reduce the dependence on physical markers. This shift makes motion capture systems more accessible for applications like gaming, sports, and robotics [48]. However, marker trajectories remain irreplaceable in domains requiring extreme precision [63].

### 5.4. Evaluation Methods

Evaluation strategies in human motion recovery research emphasize accuracy, robustness, efficiency, and practical applicability. Among all metrics, Root Mean Square Error (RMSE) is the most widely used, cited in 16 studies (34.8%), particularly in RNNs, LSTMs, and optimization-based methods [3,33]. Its sensitivity to deviation makes it a reliable indicator of reconstruction precision. In addition, accuracy metrics are frequently applied, especially in attention-based and low-rank recovery approaches, reflecting a focus on overall correctness. Joint-specific metrics, such as Mean Per Joint Position Error (MPJPE)/Joint Position Error (JPE)/and Joint Orientation Error (JOE), used in 13% of studies (e.g., [37,53]), provide detailed evaluation at the anatomical points.Moreover, metrics like Smoothness and Average Bone Length Error (ABLE) are employed in graph- and flow-based models to ensure anatomical consistency [3,38,39]. Computational metrics, including execution time, are essential in real-time scenarios; for example, in [24] reports a 21% reduction in worst-case gap-filling error and 80% speedup improvement in completion time. Qualitative methods such as visual inspection and user studies e.g., [48] assess motion realism. Frequency-domain metrics (e.g., [41]) analyze the similarity of generated sequences to ground truth data, enhancing evaluation comprehensiveness. Cross-validation and controlled noise simulations are commonly used to test generalizability and robustness [56,60]. Comparative analyses appear in over half of the studies (52.2%) to benchmark against state-of-the-art models [40]. Ablation studies (e.g., [35]) help isolate the contributions of individual components, which identifies the impact of specific neural modules. Finally, statistical tests like ANOVA and paired *t*-tests confirm significance [29], while multi-dataset evaluations ensure generalizability across motion types (e.g., [25]). In summary, current evaluation frameworks combine quantitative, qualitative, and statistical methods to ensure robust, generalizable, and application-relevant motion recovery.

### 5.5. The Most Effective Techniques in the Human Motion Recovery Field

The most effective techniques identified in the collected studies are Spatial-Temporal Transformers, Attention-LSTM networks, Graph Neural Networks (GNNs), Low-Rank Optimization and PCA-based techniques, and Generative Adversarial Networks (GANs). These methods align closely with the comparative matrix, which evaluates their effectiveness across key dimensions such as robustness, accuracy, adaptability, computational efficiency, dataset dependency, and application suitability [2,4,5,8,10,11,15,68] (see Table 3). The chosen dimensions for comparing techniques ensure a comprehensive evaluation aligned with real-world challenges and applications. Robustness addresses performance under noisy, incomplete, or occluded data conditions, which are common challenges in MoCap datasets. Accuracy is essential for reconstructing motion data that closely aligns with the ground truth, ensuring realism and reliability in biomechanics, animation, and robotics applications. Adaptability evaluates how well techniques generalize across diverse datasets and motion types, reducing the need for retraining or customization and enhancing versatility. Computational Efficiency considers resource and time constraints, making it a critical factor for real-time or resource-limited applications like live motion tracking or rehabilitation systems. Dataset Dependency reflects the practicality of methods in scenarios with limited or low-quality data, highlighting techniques that can perform well with minimal or incomplete training datasets. Finally, Use Cases tie each technique to specific applications where it excels, providing practical insights into its suitability for tasks such as gap filling, motion refinement, or occlusion recovery. These dimensions, derived from literature, provide a balanced framework for evaluating MoCap recovery techniques in academic contexts. The selection of recovery techniques depends on the specific needs of each application, including dataset complexity, resource constraints, and the level of accuracy required. Spatial-Temporal Transformers and Graph Neural Networks (GNNs) are particularly suited for handling complex datasets, offering exceptional accuracy and adaptability. Attention-LSTMs provide a practical compromise between performance and computational efficiency, making them well-suited for moderate recovery tasks. For small-scale or structured datasets, Low-Rank Optimization and PCA techniques deliver reliable and efficient solutions, while GANs play a complementary role by enriching training datasets with realistic synthetic motion sequences, indirectly enhancing recovery outcomes. Statistical evaluations highlight the effectiveness of these approaches: GNNs achieve up to a 40% reduction in marker occlusion errors, GAN-augmented datasets improve classification accuracy by 15%, and PCA-based methods maintain interpolation errors below 10% for linear motion patterns. By aligning these methods with their optimal applications, researchers can effectively address specific MoCap challenges, driving advancements in diverse fields such as animation, biomechanics, sports science, and robotics.

### 5.6. Analyzing Citation Patterns

An analysis of influential studies, based on citation number, provides a comprehensive perspective on the domain’s progression. The top five papers exemplify impactful contributions, establishing benchmarks that have shaped methodologies and advanced the field. One of the most influential works is by Alejandro et al. (2019), which has garnered 222 citations [41]. This study employs fully convolutional generators and discriminators to predict missing motion data by leveraging spatiotemporal patterns. It produces realistic and smooth recovery results that set a high standard for motion data reconstruction. Another foundational study by Holden (2018), with 116 citations [22]. It introduces a deep denoising feedforward neural network that emphasizes motion smoothness and robust skeleton configurations, making it a cornerstone in motion denoising. In more recent work by Cui and Sun (2021), cited 78 times, proposes a multitask graph convolutional network (MTGCN) that integrates spatial and temporal features to handle incomplete motion data with high accuracy [62]. Another study by Cui et al. (2021) has received 73 citations [40]. It employs temporal convolutional GANs to predict future motion based on incomplete sequences. Lastly, the study by Mall et al. (2017), with 53 citations, uses bidirectional recurrent neural networks (RNNs) with attention mechanisms to clean noisy motion data, making it a valuable contribution to noise reduction in motion capture [31]. These studies highlight the significant progression and diversification of motion capture recovery techniques and exhibit several notable similarities, highlighting key trends in the field. A central theme across these works is the reliance on deep learning architectures, such as convolutional neural networks (CNNs), recurrent neural networks (RNNs), and generative adversarial networks (GANs), which are specifically designed to handle the complex temporal and spatial dependencies inherent in motion capture data. Notably, each study leverages these powerful frameworks and introduces unique architectural innovations tailored to overcome specific challenges. For instance, integrating multitask graph convolutional networks (MTGCN) addresses spatial relations across joints, while bidirectional RNNs with attention mechanisms enhance understanding of long-term dependencies. Additionally, GAN-based approaches prioritize the generation of realistic and biomechanically plausible motion sequences, ensuring smoothness and natural continuity.

### 5.7. Limitations and Future Directions

The analysis of recovery methods for human motion data reveals several critical limitations, biases, and recurring failure cases that must be addressed to advance the field. A primary challenge is computational inefficiency: advanced techniques such as transformer-based networks and low-rank matrix recovery, while effective, demand substantial resources and remain unsuitable for real-time or large-scale deployment [46]. Closely related are biases stemming from data dependency. Many learning-based approaches, including GANs, LSTMs, and GNNs, rely heavily on large, curated datasets such as CMU or HDM05 [53,60], which skews performance toward controlled laboratory conditions and limits applicability in diverse real-world scenarios. These benchmarks lack diversity in clothing, occlusion, and multi-person interactions, restricting ecological validity and masking failure cases in dynamic environments. Failure cases are evident across methodological categories. PCA regression and inverse kinematics oversimplify trajectories under occlusion, producing unrealistic reconstructions [24,28]. GANs often collapse when noise is irregular or training data is insufficient, while LSTMs and attention-based models generalize poorly to unseen subjects or motion styles [32,50]. Transformers struggle with long consecutive gaps, GRUs saturate under irregular sequences, and GNNs fail when skeletal topology is incomplete or inconsistent. Robustness remains a persistent weakness: many models are evaluated only on synthetic noise, which fails to replicate the complexity of real-world corruption such as marker occlusion, sensor drift, motion blur, or depth ambiguity. This reliance on synthetic evaluation inflates reported performance and undermines real-world reliability [17,25]. Application specificity further restricts adaptability, as methods such as Graph-Based Normalizing Flow and MarkerNet [37,38] are designed for particular marker configurations, limiting their effectiveness in broader contexts such as markerless motion capture. Addressing these limitations requires an integrated research approach. Improving generalization through diverse and realistic datasets including varied body shapes, clothing, environments, and multi-person interactions will enhance applicability across subjects and motion types [36,39,55]. Lightweight architectures, such as efficient transformers and recurrent networks, can reduce computational demands and support real-time recovery [3,61]. Broader datasets and interdisciplinary collaboration will aid benchmark development and improve reliability [5,46]. Incorporating biomechanical constraints, for example, joint-angle limits, bone-length stability, and physics-informed priors, will ensure anatomically plausible reconstructions under occlusion. Multimodal inputs, including IMUs, depth sensors, and video, can strengthen robustness, while generative models such as GANs and normalizing flows can enrich training data [37,60]. Finally, context-aware frameworks that integrate multi-agent interactions and environmental dynamics [8,34], alongside uncertainty-aware modeling, will support more comprehensive and reliable recovery systems capable of handling complex, noisy, and unpredictable real-world scenarios.

## 6. Conclusions

This review comprehensively analyzed motion capture (MoCap) data recovery techniques, emphasizing the growing influence and suitability of artificial intelligence (AI) in overcoming the limitations of traditional approaches. Recent advancements—particularly in deep learning architectures such as transformers, graph neural networks (GNNs), and generative adversarial networks (GANs)—have demonstrated remarkable capabilities in reconstructing complex, high-dimensional motion data. These AI-based methods outperform traditional optimization and interpolation models by learning nonlinear spatial–temporal dependencies and adapting to diverse motion patterns with high accuracy. Hybrid methodologies that integrate AI with physics-based or statistical frameworks present a promising direction, combining interpretability and computational efficiency with AI’s adaptability and precision. Despite these advances, significant challenges remain, including high computational costs, dependence on large and well-annotated datasets, and limited real-world generalization. Addressing these gaps will require the development of lightweight, real-time AI architectures and the creation of unified evaluation benchmarks to ensure consistency and reproducibility across studies. Future research should also explore multimodal data fusion—integrating visual, inertial, and biomechanical signals—to enhance robustness and contextual understanding. By aligning AI-driven innovations with practical motion capture applications, the field is poised to achieve significant breakthroughs, enabling reliable, efficient, and context-aware MoCap recovery systems for biomechanics, robotics, healthcare, and immersive technologies.

## Figures and Tables

**Table 1:** Comparison between the different categories of motion capture recovery methods.

Category	Main Advantages	Main Limitations	Good For	Representative Studies
Non-Data-Driven Models	Reliable and computationally efficient. Performs well on structured, less-noisy data. Where interpretability and deterministic behaviour are important.	Limited scalability to very large or highly corrupted datasets. Struggle to adapt to unstructured or extremely noisy data and are less effective in handling complex, nonlinear motion patterns.	Well-structured and low-noise motion capture datasets; applications requiring fast processing and high interpretability, such as gap filling and trajectory smoothing in controlled environments.	[24,25,26,27,28,29,30]
Data-Driven Models	Excel at handling noisy and complex data. Adapt well to large datasets and can model nonlinear spatial–temporal dependencies. Provide high reconstruction accuracy when sufficient training data are available.	Require extensive training data and substantial computational resources. Sensitive to overfitting, domain shift, and dataset bias, especially with limited or imbalanced data. Model behaviour can be less interpretable.	Complex and noisy motion capture datasets; marker recovery and motion prediction; animation, biomechanics, and robotics applications where high precision and flexibility are required.	[17,31,32,33,34,35,36,37,38,39,40,41]
Hybrid Models	Combine the adaptability of neural networks with domain-specific or biomechanical constraints. Suitable for real-time and clinical/biomechanical applications where both accuracy and physical plausibility are important. Balance precision with interpretability and robustness.	Slightly less adaptable to highly diverse or unstructured datasets than purely data-driven approaches. Model design and integration of constraints can increase implementation and tuning complexity.	Real-time motion capture applications; biomechanical analysis with physical constraints; denoising and gap filling where domain knowledge or physics-based priors must be respected.	[42,43,44]

*Note*: The table synthesizes advantages, limitations, and application scenarios reported across the reviewed literature for each methodological category. The cited works provide representative examples rather than an exhaustive list; a more detailed analysis is presented in Section 4 and Section 5.

**Table 2:** Purpose of Techniques for Motion Data Recovery. Symbols: ✓ = applicable; ✗ = not applicable.

Techniques	Noise Removal	Gap Filling	Quality Enhancement	Studies
Bi-directional Attention Networks (BAN) with Bi-LSTM, Deep Denoising Feedforward Neural Networks, Spatial-Temporal Graph Motion Glow (STMG), Divide-and-Conquer Strategies.	✓	✓	✓	[22,31,33,35,36,37,38,39,53,54,55]
Attention-based neural networks, Bidirectional RNNs GANs and DenseNet	✓	✗	✓	[17,32,56,57,58,59]
GANs, DenseNet and SVMs	✗	✗	✓	[48,49,60]
Attention-Based Transformers, Low-Rank Matrix Restoration with Sparse Priors and Kalman Filters, Temporal Convolutional GANs (TCGAN)	✗	✓	✓	[3,25,26,27,28,29,30,34,40,41,43,45,46,50,61,62]
LSTM, GRU, FFNN, and BiLSTM, Locally Weighted PCA Regression, Regression Trees, Low-Rank Matrix Recovery, Non-Convex Optimization, kd-Tree Structures	✗	✓	✗	[24,42,44,51,52,63,64]
Graph Neural Networks combined with Temporal Transformers	✓	✓	✗	[47]

**Table 3:** Comparison of The Most Effective Techniques.

Dimension	Spatial-Temporal Transformers	Attention-LSTM (ALSTM)	GNNs	PCA	GANs
Robustness	Handles long gaps, occlusions; RMSE validated (34.8%).	Effective for mid-length occlusions; MPJPE, RMSE validated (21.7%).	Excellent for occlusions, joint connectivity; JPE, JOE validated (13%).	Struggles with noisy data; RAJE validated (6.5%).	Dependent on downstream models, F1 and AUC were validated (17.4%).
Accuracy	High precision for dynamic motions; BLE, RMSE validated (34.8%).	Reliable for moderate dynamics; RMSE validated (21.7%).	Accurate for spatial-temporal tasks; JPE, BLE validated (13%).	Limited for complex patterns; RAJE, MSE validated (6.5%).	High fidelity in augmented datasets; F1, RMSE validated (17.4%).
Adaptability	Generalizes well to unseen data; validated on large datasets (26.1%).	Good generalization via attention; MPJPE validated (13–21.7%).	Handles heterogeneous motions; NPE, BLE validated (13%).	Poor adaptability to new types; RAJE, MSE validated (6.5%).	Augments specific data scenarios; AUC validated (17.4%).
Computational Efficiency	High computational cost; execution time validated (13%).	Moderate cost; completion time validated (8.7%).	Graph construction adds complexity; validated with NPE (13%).	Highly efficient for small tasks; MSE validated (6.5%).	Resource-intensive training; F1, RMSE validated (17.4%).
Dataset Dependency	Requires large datasets; BLE, RMSE validated (26.1%).	Moderate dataset needs. MPJPE, RMSE validated (13–21.7%).	Diverse datasets required; NPE, BLE validated (13%).	Minimal dataset needs. RAJE, MSE validated (6.5%).	Effective for augmenting small datasets; AUC, F1 validated (17.4%).

## Data Availability

Data sharing is not applicable to this article as no new data were created or analyzed in this study.
